# Design, Control, and Validation of a Symmetrical Hip and Straight-Legged Vertically-Compliant Bipedal Robot

**DOI:** 10.3390/biomimetics8040340

**Published:** 2023-08-01

**Authors:** Jun Tang, Yudi Zhu, Wencong Gan, Haiming Mou, Jie Leng, Qingdu Li, Zhiqiang Yu, Jianwei Zhang

**Affiliations:** 1 School of Optoelectronic Information and Computer Engineering, University of Shanghai for Science and Technology, Shanghai 200093, China; 211240187@st.usst.edu.cn (J.T.);; 2 Institute of Machine Intelligence, University of Shanghai for Science and Technology, Shanghai 200093, China; 3 School of Health Science and Engineering, University of Shanghai for Science and Technology, Shanghai 200093, China; 4 Beijing Advanced Innovation Center for Intelligent Robots and Systems, School of Mechatronical Engineering, Beijing Institute of Technology, Beijing 100081, China; 5 Key Laboratory of Biomimetic Robots and Systems, Beijing Institute of Technology, Ministry of Education, Beijing 100081, China; 6 Yangtze Delta Region Academy of Beijing Institute of Technology, Jiaxing 314000, China; 7 Department of Informatics, University of Hamburg, 20146 Hamburg, Germany

**Keywords:** bipedal walking, legged robot design, DCM

## Abstract

This paper presents the development, modeling, and control of L03, an underactuated 3D bipedal robot with symmetrical hips and straight legs. This innovative design requires only five actuators, two for the legs and three for the hips. This paper is divided into three parts: (1) mechanism design and kinematic analysis; (2) trajectory planning for the center of mass and foot landing points based on the Divergent Component of Motion (DCM), enabling lateral and forward walking capabilities for the robot; and (3) gait stability analysis through prototype experiments. The primary focus of this study is to explore the application of underactuated symmetrical designs and determine the number of motors required to achieve omnidirectional movement of a bipedal robot. Our simulation and experimental results demonstrate that L03 achieves simple walking with a stable and consistent gait. Due to its lightweight construction, low leg inertia, and straight-legged design, L03 can achieve ground perception and gentle ground contact without the need for force sensors. Compared to existing bipedal robots, L03 closely adheres to the characteristics of the linear inverted pendulum model, making it an invaluable platform for future algorithm research.

## 1. Introduction

Humanoid robots can effectively navigate narrow paths and obstacles such as stairs, outperforming wheeled and tracked robots in adaptability and energy efficiency [1]. The core research in the field of humanoid robotics revolves around bipedal locomotion, with 3D walking, dynamic stability, energy efficiency, and cost constituting key components of practical application. Over the years, efforts on the part of industry have concentrated on enhancing the walking abilities of humanoid robots [2]. Owing to continuous advancements in structural design, motor technologies, and gait algorithms, humanoid robots such as Atlas which employ an active walking strategy have demonstrated exceptional locomotive performance [3]. In recent years, significant progress has been made in research on bipedal robots involving various design and control methods. Among them, planar compass bipedal robots and driven bipedal robots have garnered considerable attention as representative design approaches. Planar compass bipedal robots achieve gait control through passive dynamic principles and simple control mechanisms, resulting in a simplified structure and control system. On the other hand, driven bipedal robots influence their gait through active control of the trunk, providing higher autonomy and adaptability. Chen et al. developed a stair-climbing bipedal robot utilizing telescopic legs and impulsive propulsion for gait control [4]. Meng and Song presented a challenge terrain walking bipedal robot for complex terrains [5]. Zhou et al. explored the gait control and dynamic walking characteristics of a semi-passive compass-like bipedal robot using impulsive propulsion [6]. Added et al. proposed a trajectory tracking-based control method to control the chaotic behavior of compass-like robots, further analyzing passive dynamics and chaotic control of compass-like bipedal robots [7,8]. Additionally, C. Chevallereau and E. R. Westervelt provided important guidance on bipedal robot modeling, design, and gait synthesis, delving into crucial issues in the field of bipedal robots [9,10]. Their contributions are of significant theoretical and practical significance in advancing the development and application of bipedal robot technology. A key area of future research is amalgamation of the respective strengths of active and passive walking robots to develop humanoid robots that are energy efficient and dynamic while maintaining cost-effectiveness.

Presently, traditional fully-articulated active robots grapple with numerous challenges associated with the number of joints, dynamic performance, and rigidity. These factors contribute to the complexity of their control models, deviating significantly from the inverted pendulum model and hindering research on dynamic walking algorithms. Therefore, it is crucial to reduce the number of actuators and improve the dynamic performance of robots.

Numerous research teams are currently engaged in the exploration of under-actuated bipedal robots. One such example is the robot Cassie, which, through the reduction of rotational degrees of freedom in ankle joints and the utilization of ten actuators, has been successful in achieving outdoor locomotion [11]. Another robot, Blot, manages to attain three-dimensional dynamic balanced walking with only six actuators by refining the design of the hip and ankle joints, showcasing remarkable anti-interference capabilities [12]. Additionally, the robot SLIDER, featuring a distinctive kneeless leg design and ten degrees of freedom, is an ultra-lightweight and cost-effective bipedal walking robot [13].

Extensive research has been conducted on under-actuated passive walking robots in this area. In 2011, Cornell University’s Ranger robot achieved continuous walking for 65 km using only four actuators [14]. In 2015, Xingzhe No. 1, developed by Chongqing University of Post and Telecommunication in China, completed continuous walking for 134 km around a playground using only two actuators [15].

We consider the determination of the minimum number of actuators required for achieving omnidirectional walking in bipedal robots to be a topic worthy of discussion. Currently, publicly available bipedal robots with omnidirectional walking capabilities require a minimum of six actuators. On the other hand, passive walking robots can achieve stable forward walking with only one actuator. Therefore, theoretically, by adopting a split design scheme for the hip joints in the sagittal and lateral directions it is possible to achieve omnidirectional walking in bipedal robots using only four actuators. However, for better balance in bipedal robots, additional actuators may be required.

In this research, we created L03, a bipedal robot that incorporates symmetrical design principles characteristic of passive walking robots. This innovative design requires only five actuators, two for the legs and three for the hips. With a height of 0.94 m and a weight of 5.5 kg, L03 is an ultra-lightweight, kneeless, under-actuated, high-dynamic, and cost-effective bipedal walking robot. The swing leg has a minimal weight of 650 g. L03 utilizes DCM-based motion trajectory planning, concentrating on the center of mass and foothold, achieving stable forward and lateral movement in the actualization of a 3D bipedal walking robot.

The remainder of this paper is organized as follows: Section 2 introduces the mechanical system design related to the robot’s joint; Section 3 presents an analysis of the robot’s lateral kinematics; Section 4 discusses the trajectory planning algorithm for the bipedal robot based on DCM; Section 5 demonstrates through experiments that the under-actuated biped robot can maintain stable lateral and forward 3D walking; finally, Section 6 concludes the research and discusses potential directions for future work.

## 2. Design Overview

**Figure 1:**
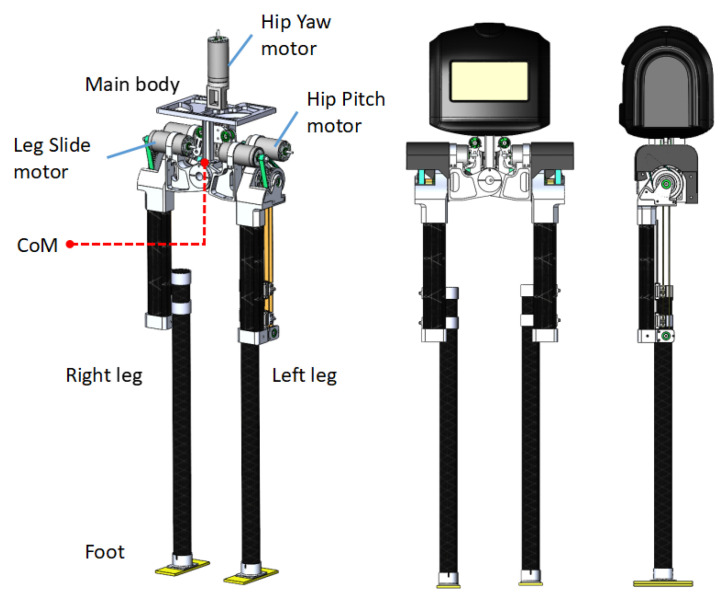
A model of the L03 robot; it has five degrees of freedom and straight legs crafted from carbon fiber-reinforced polymer. Designed to be ultra-lightweight, it weighs only 650 g and possesses a symmetrical hip structure.

The mechanical structure design of a bipedal robot must fulfill the requirements of high stiffness, high flexibility, low inertia, and low damping [16,17]. As illustrated in Figure 1, the L03 robot is designed as a kneeless bipedal walker consisting of a pair of feet, a pair of legs, and a main body frame. This robot features five joints and is intended to be ultra-lightweight, highly dynamic, and cost-effective, making it suitable as a research platform for bipedal locomotion. L03’s under-actuated design scheme adopts a lateral symmetric hip mechanism that realizes the platform functionality of an omnidirectional bipedal robot using only five drive motors. Its most prominent advantage is the maximized reduction of motor quantity and the elimination of knees, consequently minimizing the robot’s weight and the complexity of the leg design. To fully capitalize on the benefits of the kneeless design (which reduces the leg mass) and better adapt to the requirements of passive walking algorithms [18], the primary design objectives of L03 include: (1) lightweight, high stiffness, and compact joints; (2) concentration of mass as close to the main body frame as possible; (3) low development cost; and (4) a design that is easy to maintain.

### 2.1. Hip Structure Design

The design of a bipedal robot’s hip joint necessitates the incorporation of six degrees of freedom (DoF), which include hip roll, hip yaw, and hip pitch. Currently, there are four dominant design strategies. (1) Serial drive: this method connects the joints in series in the order of roll, yaw, and pitch. This straightforward and simple arrangement is currently the most prevalent [1]. (2) Serial-parallel drive: in this approach, the hip roll joint serves as the main joint, and is linked to the yaw and pitch joints in parallel. Typical parallel techniques involve using connecting rods, rope drives, and umbrella teeth [19]. (3) Parallel drive: this strategy designs the hip as a spherical joint, merging the three DoFs at a single point. Although this maximizes motion inertia, its intricate design makes it less commonly utilized [20]. (4) Underactuated design: this strategy eliminates the roll joint, maintaining only the yaw and pitch. While this results in a loss of in-place rotation ability, it simplifies the design, improves the robot’s rigidity, and is particularly valuable for gait algorithm research [21].

**Figure 2:**
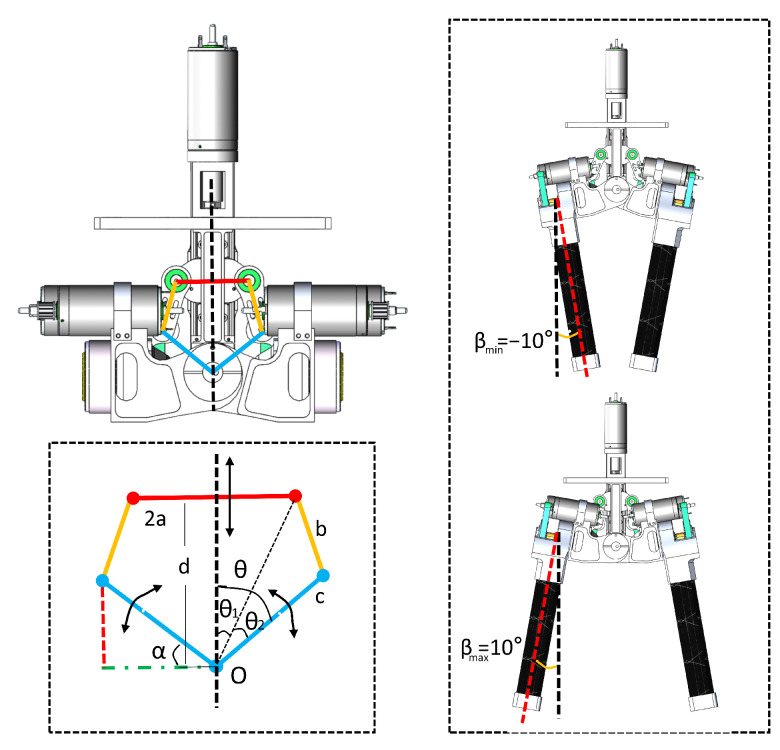
Overall structure design of the robot hip.

To enhance the energy efficiency of the robot further, this paper introduces a two-in-one bisection design scheme that employs a symmetrical four-bar linkage mechanism to achieve lateral hip bisection swing. The design principle is illustrated in Figure 2. Let the length of the crossbar be 2a, the length of the pull-up rod be *b*, the length of the pull-down rod be *c*, and the height of the vertical rod be *d*. The angle, denoted as θ, between the pull rod and the vertical rod is decomposed into $\theta_{1}$ and $\theta_{2}$ using trigonometric principles. Following this, we derive the values of $\theta_{1}$ and $\theta_{2}$ employing trigonometric equations, as delineated in Equations (1)–(3). Considering the lateral hip joint’s relative fixity to the pull-down rod *c*, it can be inferred that any variation in the angle θ is synonymous with the change in the swing angle of the lateral hip joint.

(1) $$\theta_{1}=\arctan\frac{a}{d}$$

(2) $$\theta_{2}=\arccos\frac{a^{2}+d^{2}+c^{2}-b^{2}}{2c\sqrt{a^{2}+d^{2}}}$$

(3) $$\theta =\theta_{1}+\theta_{2}.$$

**Figure 3:**
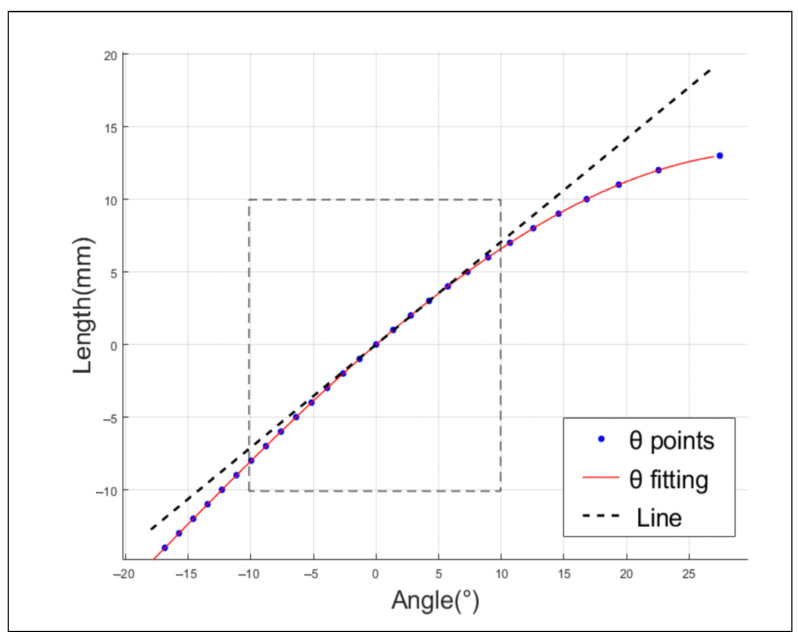
Linearity between the lateral hip joint and the drive motor.

To satisfy the design range of motion for the lateral swing of the hip, which is [−10°, 10°], the crossbar *a* is set to 25 mm, the pull-up rod *b* is 35 mm, and the pull-down rod *c* is 45 mm. The vertical link *d* ranges from 47.7 mm to 77.7 mm. A simulation curve is then generated using both forward and inverse solutions. As illustrated in Figure 3, the linearity reaches 90% when the range of lateral θ is [−10°, 10°]. This particular set of parameters, which takes into account linearity, transfer efficiency, and speed range, aligns with the design requirements.

As illustrated in Figure 1, the robot body and the linear module are securely assembled to create a platform that houses hardware circuits, batteries, gyroscopes, and other components. The hip assembly of the L03 robot is composed of five Maxon DC motors: one Hip Yaw motor, two Hip Pitch motors, and two Leg Slide motors. The Hip Yaw motor, positioned centrally within the main body, functions as the initial joint. The lateral hip joint employs a symmetrical four-bar linkage mechanism, with the base of the KK ball screw slide table acting as the vertical supporting rod *d*. The movable nut seat of the screw slide table, in conjunction with the crossbar fixture, forms the crossbar *a*. The utilized ball screw, which has a diameter of 6 mm and a lead of 2 mm, accomplishes a travel distance of 30 mm. To minimize rotational inertia, both Hip Pitch motors and Leg Slide motors are symmetrically and adjacently mounted on the respective left and right sides of the pull-down rod *c*. For instance, the left Hip Pitch motor, integrated with a harmonic reducer within the pull-down rod *c*, facilitates the hip’s anterior/posterior swing. The Leg Slide motor, positioned parallel to the reducer, employs a timing belt for power transmission, sequentially driving the leg extension. Finally, the pull-up rod *b* forms hinge connections with rods *a* and *c*, thereby completing the four-bar linkage structure.

The Hip Yaw motor, linked to the screw slide table, governs the movement of the screw nut slider, attaining a maximum speed of 155 mm/s. As illustrated in Figure 3, the displacement of crossbar a by 20 mm induces a 20° movement in the Lateral Hip joint. As a result, the Lateral Hip joint achieves an approximate rotational speed of 155°/s at its peak. The Hip Pitch motors are integrated with the HD harmonic reducer via a timing belt. This harmonic reducer delivers a maximum output speed of 540°/s, a rated output torque of 5.4 Nm, and a maximum torque of 56 Nm. Detailed specifications of the robot are provided in the ensuing Table 1.

### 2.2. Leg Structure Design

In the realm of bipedal robots, leg design is a pivotal aspect of the overall design process. The primary goal, as highlighted in the literature, is to minimize the leg’s moment of inertia [16]. A prevalent strategy is the strategic placement of heavier drive components, such as motors and reducers, at the thigh’s base or fixed at the hip joint. Through transmission mechanisms such as connecting rods, pull ropes, and synchronous belts, power is transferred to the knee and ankle joints, thereby reducing the leg’s moment of inertia. Concurrently, the use of lightweight materials such as aeronautical aluminum alloy and carbon fiber can further decrease the leg’s mass and inertia. Robots such as Cassie, Bolt, and Kangaroo Robot have successfully utilized this design strategy to reduce leg inertia [19,22,23]. Bipedal robot leg designs can be broadly classified into two categories, namely, those with a knee joint and those without. While the majority of bipedal robots prefer knee joint designs due to their biomimetic attributes, there are numerous compelling examples of kneeless designs, with Google’s Schaft Robot being representative [13], provides a clear comparison of both designs. Generally, for a debugging robot platform a kneeless model might be more appropriate. Considering the control algorithms and cost, the L03 robot employs a telescopic kneeless design strategy, sacrificing biomimetic effects to enhance the robot platform’s performance. This strategy aims to maximize the reduction in leg mass and moment of inertia and increase leg flexibility to accommodate the simplest model algorithm.

**Figure 4:**
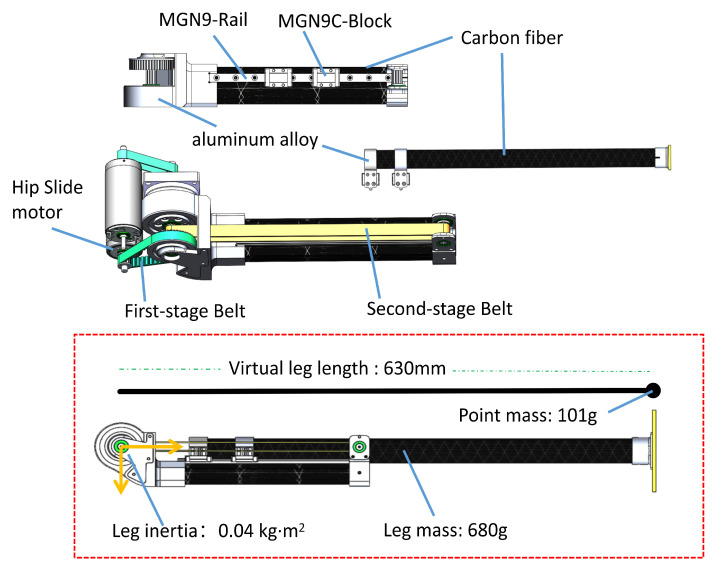
Overall structure design of the robot leg.

As illustrated in Figure 4, the conversion of the joint actuator’s rotational movement into the leg’s linear movement enables the implementation of the leg sliding mechanism. To achieve an ultra-light leg design, the drive motor is fixed at the hip and power is transmitted by a synchronous belt. The L03 robot utilizes a two-stage synchronous belt to drive the lower leg’s telescopic movement. The first-stage belt system allows for a six-fold reduction in the drive motor speed, simultaneously amplifying the torque by the same factor and transmitting the drive to the upper leg’s root. The second-stage belt system, forming a closed loop, spans from the root to the upper leg’s extremity. The lower leg, which is attached to the second-stage belt, achieves vertical movement in harmony with the motion of the second-stage belt. Carbon fiber tubes are primarily used in order to balance rigidity and lightness in the sliding joint, supplemented with a small amount of aluminum alloy parts for fixation. To ensure precise leg telescoping, a linear rail is used to connect the upper and lower legs. Through this design, the weight of an individual leg is reduced to 680 g, while the moment of inertia of the leg is only 0.04 kg/m^2^. This inertia is equivalent to the inertia produced by a mass point of 101 g swinging around a radius of 630 mm. Figure 4 illustrates the design details of the belt drive solution for the leg along with the ultra-light leg’s weight and moment of inertia. In summary, the leg design of the L03 robot offers several key features:

1. Due to the unique capabilities of the kneeless telescopic leg design, the robot is able to directly perceive changes in ground contact forces without the need for force sensors, meaning that when the robot’s foot makes contact with the ground, the resulting reaction forces are directly transmitted to the leg motors, enabling the detection of ground contact signals by monitoring changes in electrical currents;
2. The dynamic model closely aligns with a simplified mathematical model, and leg telescoping does not introduce anterior–posterior interference;
3. The kneeless telescoping leg exhibits high linearity;
4. Compared to the knee joint approach, the straight leg telescoping joint is more energy-efficient.

## 3. Kinematics Analysis

Due to their inherent complexity, bipedal robots frequently rely on simplified models such as the linear inverted pendulum (LIP) and spring loaded inverted pendulum (SLIP) for studying gait algorithms [24,25]. These models, while simplifying the robot’s body to a point mass and neglecting the effects of leg mass and body posture, offer significant insights into the fundamental properties of bipedal robot movement.

During the walking process of a bipedal robot, the motion of its center of mass (COM) is non-steady state, necessitating ongoing adjustments in body posture and foot placement to maintain balance. The DCM is a vector defined in terms of the COM’s position and velocity, and is instrumental in characterizing the robot’s dynamic behavior and stability.

The DCM is defined as:

(4) ξ=x+1/ω×dx/dt

where ξ denotes the DCM position, *x* represents the position of the COM, dx/dt signifies the velocity of the COM, and ω is a specific time constant, typically positive, primarily influenced by the robot’s dynamic parameters and control strategy.

This formula elucidates the way in which the DCM position reflects a balance between the current COM position and an anticipated future COM position, with 1/ω×dx/dt signifying the latter. Therefore, by manipulating the DCM we can influence the future position of the robot’s COM, allowing us to subsequently govern the robot’s motion trajectory and stability.

Utilizing the DCM model, we can ascertain the values of *x* and ξ at any point in time [25]. Motor drive control is essential in facilitating the robot’s movement according to this relationship and to guarantee stable walking, necessitating a kinematic analysis. The L03 bipedal robot proposed in this study features a simplistic mechanical structure with lightweight legs and low rotational inertia, aligning well with the LIP model’s characteristics. Its structural simplicity enables swift derivation of interjoint relationships through geometric principles. Using these principles, we can effortlessly calculate the lengths of the legs, the motion angles of the hip joints, and the motion angles of the lateral joints.

**Figure 5:**
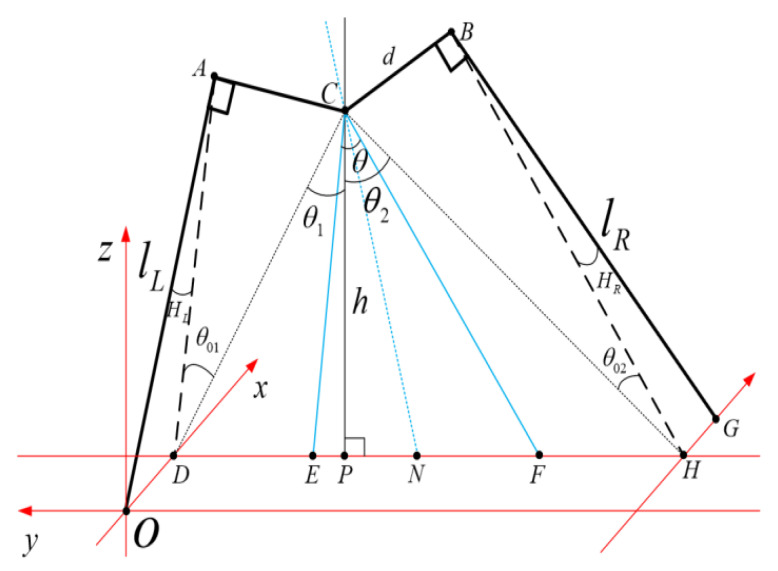
Spatial schematic diagram of the kinematics analysis.

Figure 5 illustrates the Cartesian coordinate system established at the robot’s ground contact point *O*. Contact points $D(x_{d},y_{d},z_{d})$ and *H* correspond to the virtual leg, while *O* and $G(x_{g},y_{g},z_{g})$ represent those of the real leg. The coordinate origin is denoted by O(0,0,0). The robot’s COM is defined as $C(x_{c},y_{c},z_{c})$. *A* and *B* indicate hip vertices, with AC being equal to BC, both of length d. Furthermore, AC and BC are perpendicular to the robot’s left and right legs, respectively, θ denotes the robot’s bipartition angle, CN represents the bisector line of θ, and CE is parallel to AD, while CF is similar to BH. The rotation angles of the left and right virtual legs are denoted as $H_{L}$ and $H_{R}$, respectively, $l_{L}$ and $l_{R}$ denote the lengths of the left and right legs, respectively, and CP represents the height *h* from the robot’s COM to the ground. The specific derivations and calculation processes are detailed as follows:

(5) $$\theta_{1}=atan\left(y_{c}/h\right)$$

(6) $$\theta_{2}=atan\left(\left(y_{g}-y_{c}\right)/h\right)$$

(7) $$\theta_{01}=asin\left(d/\sqrt{y_{c}^{2}+h^{2}}\right)$$

(8) $$\theta_{02}=asin(d/\sqrt{{\left(y_{g}-y_{c}\right)}^{2}+h^{2}}$$

(9) $$\theta =\frac{\left(\theta_{1}+\theta_{2}-\theta_{01}-\theta_{02}\right)}{2}$$

(10) $$AD=d/\tan\theta_{01}$$

(11) $$H_{L}=atan\left(x_{d}/AD\right)$$

(12) $$l_{L}=AD/\sin H_{L}$$

(13) $$BH=d/\tan\theta_{02}$$

(14) $$H_{R}=atan\left(x_{f}/BH\right)$$

(15) $$l_{R}=BH/\sin H_{R}$$

Utilizing (9), we can determine the requisite rotation angle θ for the lateral joint, while (11) and (12) enable us to ascertain the left leg’s swing angle $H_{L}$ and length $l_{L}$. In a similar fashion, by applying (14) and (15) we can discern the swing angle $H_{R}$ and length $l_{R}$ of the right leg motor. Consequently, the forward and inverse kinematic solutions between the footfall points and the robot’s five joints are established for a comprehensive kinematics analysis of the L03 robot.

## 4. Control Principle

### 4.1. LIP Dynamics and DCM Derivation

**Figure 6:**
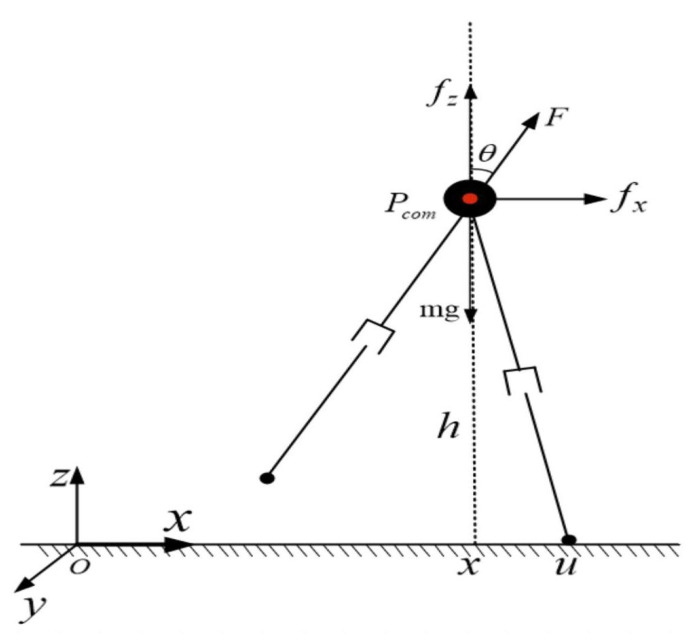
Planar inverted pendulum model.

Due to their inherent complexity bipedal robots frequently utilize simplified models such as the LIP and SLIP for the study of gait algorithms [26]. The LIP model comprises a COM and two lightweight retractable legs located in the sagittal plane. The L03 bipedal robot proposed in this study aligns well with the LIP model’s characteristics owing to its simple mechanical structure, lightweight legs, and low rotational inertia. When traversing flat ground, the COM of the inverted pendulum is assumed to move solely horizontally, disregarding any vertical movement. Thus, if the legs were to be indefinitely extended, the COM would perpetually travel along a horizontal line [27]. The vertical component Fcosθ of the telescopic force *F* exerted on the leg equals the magnitude of gravity in the opposite direction. Conversely, the COM’s horizontal movement is governed by the horizontal component Fsinθ. The height $z_{c}$ of the COM of the biped robot is fixed at 0.76 m, while the leg length is adjustable within the range of 0.58 m to 0.68 m. The forward motor modulates the legs’ forward movement (action in the *x* direction), while the lateral motor regulates their lateral movement (action in the *y* direction). The attitude angle remains symmetrical along the line passing through the COM. The ankle does not require moment input. Figure 6 illustrates this simplified model.

In the two-dimensional x−z plane, $P_{com}$ is the COM position coordinate, *u* is the contact position coordinate, and the y−z plane is similar. Let $z_{c}$ remain unchanged, and let Fcosθ=mg and $\omega =\sqrt{\frac{g}{z_{c}}}$.

Then the state space expression is

(16) $$\left[\begin{array}{c} \dot{x}\\ \dot{v} \end{array}\right]=\left[\begin{array}{cc} 0 & 1\\ \omega^{2} & 0 \end{array}\right]\left[\begin{array}{c} x\\ v \end{array}\right].$$

According to the LIP kinetic model, a specific contact point can be calculated to stop bipedal movement, at which the system’s kinetic energy equals 0. The feet are completely still, which is called caption point (CP) or the divergent component of motion [28]. According to the definition of the DCM, we can obtain the following Formula (17):

(17) $$p=x\left(t\right){|}_{t\to \infty }=cosh\left(\omega t\right)x_{0}+\frac{1}{\omega }sinh\left(\omega t\right){\dot{x}}_{0}.$$

According to the nature of the transfer function, the system transfer function has a pole of the right half-plane, the system is not asymptotically stable, and the DCM is defined according to the steady component:

(18) $$\xi =x\left(t\right)+\frac{\dot{x}\left(t\right)}{\omega }.$$

Then, we can obtain the analytical solution of ξ:

(19) $$\xi \left(T\right)=e^{\omega \left(T-t\right)}\left(\xi \left(t\right)-u\right)+u.$$

It can be seen from (19) that the *x* tracking ξ process is stable, while the ξ following *u* process is unstable; thus, ξ is called the DCM. Because the *x* following ξ process is stable, the robot can swing stably as long as ξ does not diverge during bipedal gait planning.

### 4.2. DCM Trajectory Planning

In this paper, *x* and ξ are used as the state variables of the system; the contact point coordinate *u* is planned based on the given step size and the number of steps. The contact point $u_{k}$ refers to the coordinates of the contact between the sole of the foot and the walking plane when the gait is switched at step *k*. The centroid coordinates obtained by gyroscope are calculated using the kinematics solution. The initial value of ξ for step k+1 equals the final value of step *k*. When the robot walks continuously, $\xi_{k+1}$ at the end of the last step aligns with $u_{k}$, resulting in a zero error. The previous step’s $\xi_{k}$ is inverted, allowing for subsequent planning of the COM motion trajectory $x_{k}$ through $\xi_{k}$. Equation (20) presents the system dynamics:

(20) $$\left[\begin{array}{c} \dot{x}\\ \dot{\xi } \end{array}\right]=\left[\begin{array}{cc} -\omega & \omega \\ 0 & \omega \end{array}\right]\left[\begin{array}{c} x\\ \xi \end{array}\right]+\left[\begin{array}{c} 0\\ -\omega \end{array}\right]u.$$

#### 4.2.1. Forward Walking

**Figure 7:**
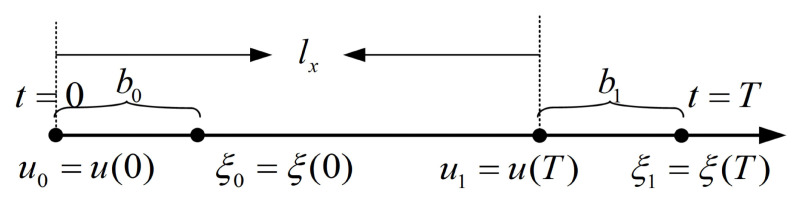
Forward walking mode.

Forward walking pertains to the robot’s locomotion within its sagittal plane. The associated model is depicted in Figure 7. The variable $b_{k}$ represents the discrepancy between the divergent element of movement, denoted as ξ, and the contact point *u*. The predetermined step length is $l_{x}$, while *T* signifies the walking cycle. The initial value of ξ for step k+1 corresponds to the terminal value of ξ for step *k*, with $b_{0}$ equating to $b_{1}$. In this way, the bipedal robots’ forward walking trajectory can be planned.

From (23), the analytical solution of the linear inverted pendulum model is known. When t=0, we have

(21) $$b_{0}=\xi \left(0\right)-u_{0},$$

(22) $$\xi \left(T\right)=e^{\omega T}b_{0}+u_{0}.$$

From the relationship between *u* and *a*, it can be inferred that for t=0, we have

(23) $$\xi \left(T\right)=u\left(T\right)+b_{0}.$$

The above equation can be converted (23):

(24) $$b_{0}+u\left(T\right)=e^{\omega T}b_{0}+u_{0}$$

Then, we can solve for (25):

(25) $$b_{0}=\frac{u\left(T\right)-u_{0}}{e^{\omega T}-1}=\frac{l_{x}}{e^{\omega T}-1}.$$

The real-time walking pattern generation method based on the decomposition of the LIP dynamics can be divided into two parts, “convergence” and “divergence”. The divergence part aims to bring the motion of the bipedal robot’s center of mass to a stop for a specific foothold, where the system’s kinetic energy becomes zero. As *t* approaches infinity, the horizontal coordinate of the center of mass at time $t_{k}$ is the same as the horizontal coordinate of the contact point *p*. The body remains upright in a static state with zero kinetic energy, and the expression for *p* is obtained as shown in (16). By applying the Laplace transform to (16), we can define ξ based on the stable part. The process of tracking ξ is stable, while the process of tracking *p* with ξ is unstable. Therefore, ξ is referred to as the motion DCM. In bipedal gait planning, because the process of tracking ξ is stable, a constraint relationship is established between ξ and *u* as long as ξ does not diverge. The usual approach is to specify ξ and *u* as being equal, allowing the robot to oscillate stably. Making $b_{1}$ equal to 0 and reversing $\xi \left(t\right)$ by $\xi \left(T\right)$, we have

(26) $$u\left(T\right)=\xi \left(T\right)-b_{1},$$

(27) $$\xi \left(T\right)=e^{\omega \left(T-t\right)}\left(\xi \left(t\right)-u_{0}\right)+u_{0}.$$

According to (27), we can obtain the value of $\xi \left(t\right)$ at each moment, which allows us to discretize $\xi \left(t\right)$ as follows:

(28) $$\xi_{k}=e^{\omega \left(T-t\right)}\left(\xi_{k+1}-u_{k}\right)+u_{k}.$$

According to the DCM principle, we set the last step’s equal $b_{1}$ to 0, that is, $u\left(T\right)$ is equal to $\xi \left(T\right)$ and $u\left(T\right)$ is the foot placement point that was pre-planned based on the step length. By reverse calculation using $\xi \left(T\right)$, we can determine $\xi \left(t\right)$. When $u_{0}=0$, representing the starting moment as the zero point, we can obtain the following equation from Formula (27):

(29) $$u\left(T\right)=e^{\omega \left(T-t\right)}\xi \left(t\right)-b_{0}.$$

Generating a reference trajectory for the DCM requires a set of pre-planned footstep coordinates, denoted as *u*. By designing the walking sequence of the bipedal robot for consecutive steps with a specified step length, the moment ξ coincides exactly with the *u* point, resulting in an error of 0. Then, by utilizing (28), we can solve for ξ(k+1). Through ξ(k+1), we can deduce the previous moment ξ(k) and, using the forward and inverse kinematics, calculate the next moment x(k+1), which represents the relationship between the foothold and the center of mass position. This ensures that ξ remains non-divergent throughout the entire walking gait of the bipedal robot.

The relationship between the motion divergence component ξ and the footstep coordinates *u* is divergent, as described by (28). To constrain this divergence relationship, we set the last step’s footstep coordinates to be equal to ξ, resulting in an error of 0 between ξ and *u*. Then, we obtain the footstep coordinates *u* for each step and use kinematic relationships to solve for the center of mass position *x*. The relationship between the motion divergence component ξ and the center of mass position *x* is established. Then, we solve $x_{k+1}$ via $\xi_{k}$:

(30) $$x_{k+1}=e^{-\omega T}\left(x_{k}-\xi_{k}\right)+\xi_{k}.$$

#### 4.2.2. Lateral Walking

**Figure 8:**
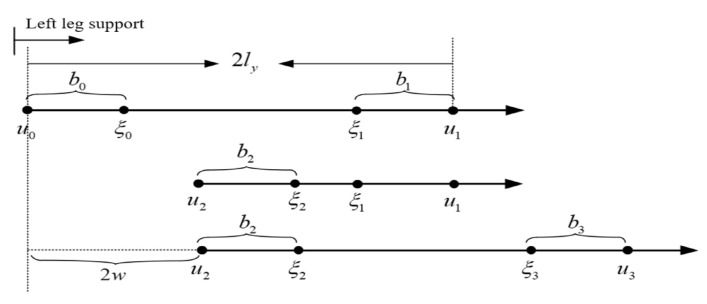
Lateral walking mode.

Lateral walking describes the movement of a bipedal robot striding sideways. If the robot shifts to the right, the right leg initiates the movement with a step to the right, followed by the left leg stepping towards the right. This sequence constitutes two steps for a single lateral shift. An identical procedure is followed for leftward motion. In this paper, we focus on DCM trajectory planning, using the rightward shift as an illustrative example; the corresponding walking model is depicted in Figure 8.

As shown in Figure 8, $2l_{y}$ is the width of the legs, 2w is the lateral shift distance, and $b_{k}$ is the difference between ξ and *u* according to the walking characteristics of lateral movement; as $b_{0}$ is equal to $b_{2}$, we have

(31) $$\left\{\begin{array}{c} b_{0}=b_{2}\\ \xi_{1}=e^{\omega T}b_{0}+u_{0}\\ \xi_{2}=e^{\omega T}b_{1}+u_{1}\\ b_{1}=\xi_{1}-u_{1} \end{array}\right.$$

(32) $$u_{2}-u_{1}=2w-2l_{y}$$

(33) $$b_{1}=e^{\omega T}b_{0}-2l_{y}$$

(34) $$b_{0}=e^{\omega T}b_{1}+2l_{y}-2w.$$

By solving (31) and (32), we can obtain

(35) $$b_{0}=\frac{2l_{y}}{e^{\omega T}+1}+\frac{w}{e^{\omega T}-1}$$

(36) $$b_{1}=\frac{-2l_{y}}{e^{\omega T}+1}+\frac{w}{e^{\omega T}-1}$$

meaning that (33) and (34) can be written as (35)

(37) $$b_{y}=-sp\cdot \frac{2l_{y}}{e^{\omega T}+1}+\frac{w}{e^{\omega T}-1}$$

where $sp=\left\{\begin{array}{cc} 1, & leftleg\\ -1, & rightleg \end{array}\right.$. According to the difference $b_{k}$ between ξ and *u*, the relationship between the contact point *u* and ξ is shown in Formula (36):

(38) $$u\left(T\right)=e^{\omega T}\xi \left(t\right)+b_{y}.$$

## 5. Simulations and Experiments

**Figure 9:**
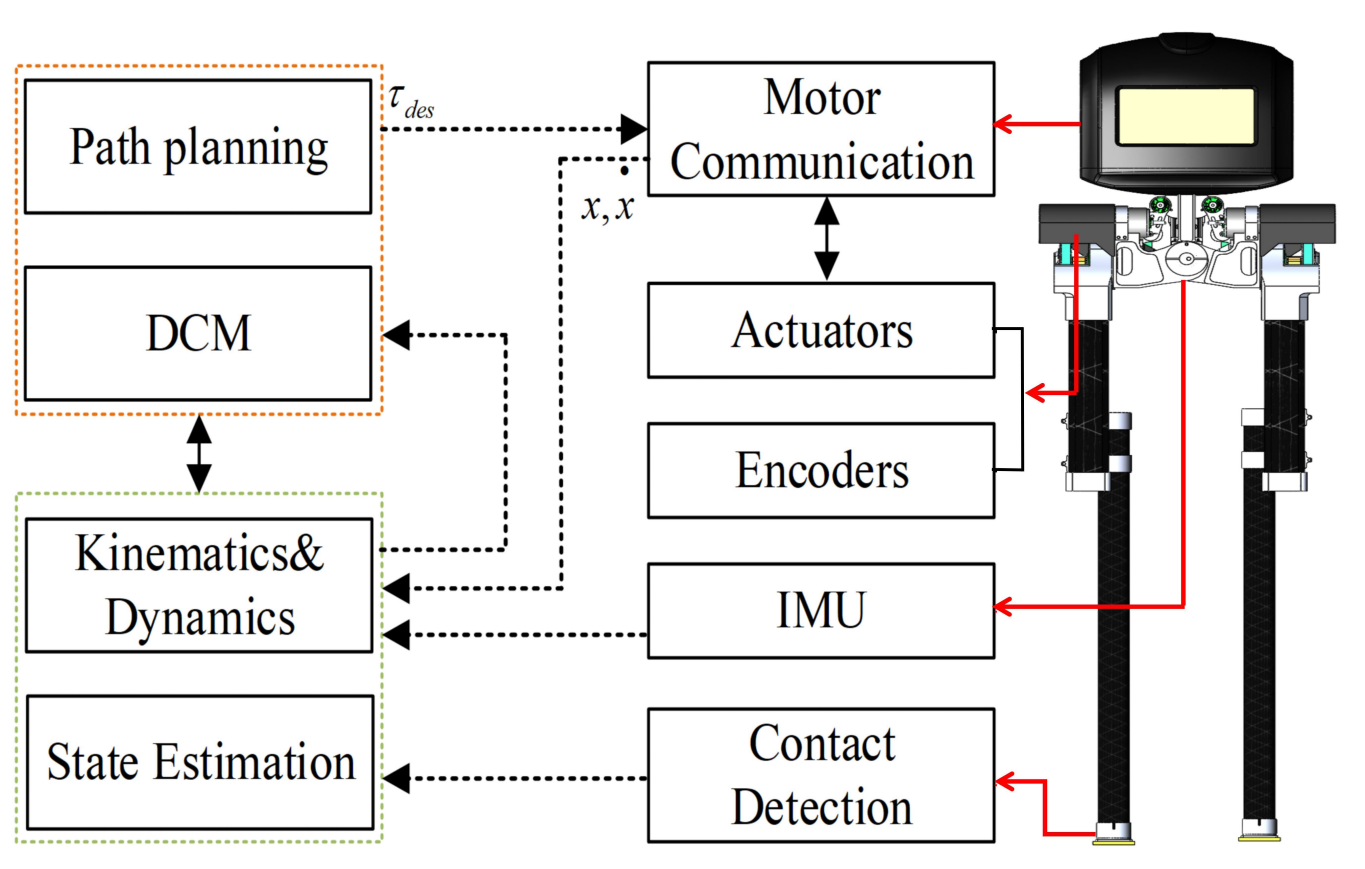
Control structure of the robot.

In gait control of bipedal robots, gait optimization is typically performed to enhance the robustness of the control system, aiming to achieve greater stability margin and lower energy consumption. In this study, we conducted walking stability verification using a bipedal robot design based on the DCM algorithm, addressing trajectory deviation issues caused by factors such as model error and external disturbances. The robot control algorithm proposed in this paper is illustrated in Figure 9. It utilizes a gyroscope to obtain the robot’s center of mass position and employs forward and inverse kinematics to calculate the foot placement position, enabling the detection of foot contact. Data are processed using an invariant Kalman filter and then passed to the DCM algorithm for trajectory planning of the robot’s foot placement. By integrating motor control components such as encoders and actuators, the algorithm significantly enhances the robustness of robot locomotion.

### 5.1. Sideways Walking

**Figure 10:**
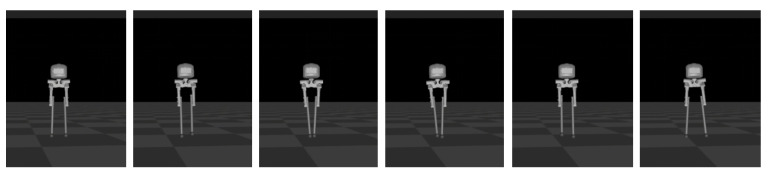
Screenshots of the L03 robot’s sideways walking in the simulation experiment.

**Figure 11:**
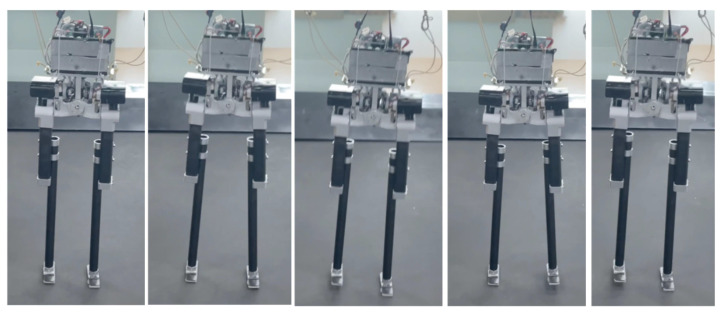
Screenshots of the actual L03 robot’s lateral walking experiment.

Sideways walking refers to the lateral movement of a robot during locomotion. In this study, we applied a control algorithm for sideways walking to the L03 robot and conducted an analysis of its stability during the walking process. To evaluate the stability of the robot in sideways walking, both simulation and physical experiments were performed to assess its performance. Figure 10 and Figure 11 illustrate the sideways walking process of the robot in the simulation and physical experiments, respectively. The experimental results demonstrate the robot’s ability to achieve stable sideways walking.

**Figure 12:**
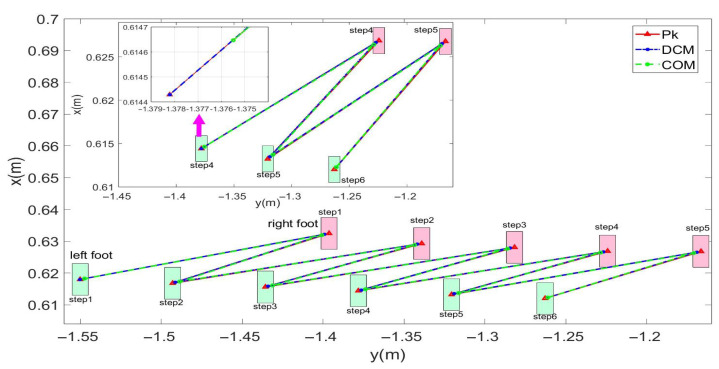
Simulation data for the L03 robot’s lateral walking.

**Figure 13:**
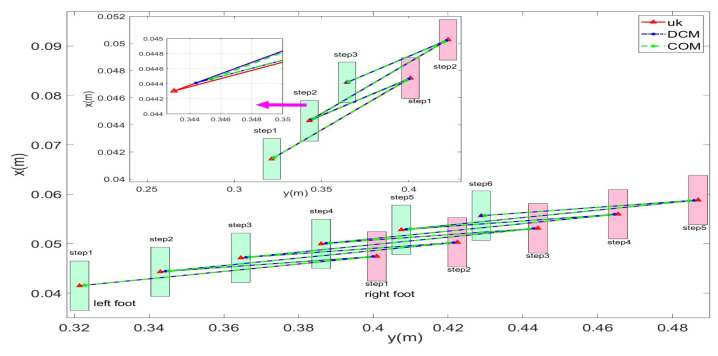
Experimental data for the L03 robot’s lateral walking.

**Figure 14:**
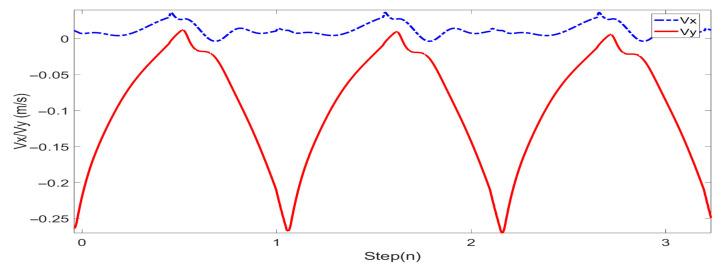
COM velocity profile of the L03 robot’s lateral walking during the simulation.

**Figure 15:**
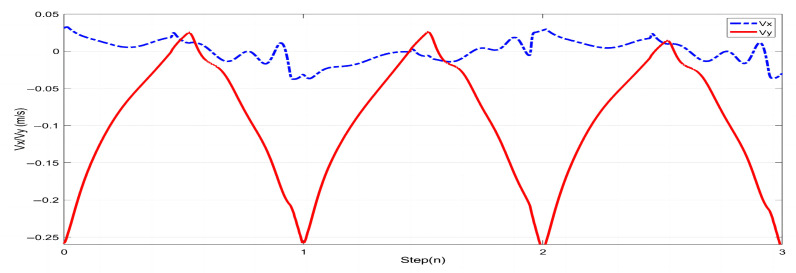
COM velocity profile of the L03 robot’s lateral walking during the experiment.

In this study, experiments on sideways walking of the robot were conducted in both the Mujoco environment and in real-world scenarios. The results of the simulations and physical experiments are shown in Figure 12, Figure 13, Figure 14 and Figure 15. A speed of 0.3 m/s and a step length of 0.06 m were set in the Mujoco simulation experiment, while in the real-world walking experiment a speed of 0.3 m/s and a step length of 0.02 m were used. All experiments were conducted on flat ground. Figure 12 and Figure 13 depict the walking gaits of the robot in the simulation and physical experiments, respectively. In the graphs, the red line represents the actual footstep position trajectory during sideways motion, the blue line represents the footstep position trajectory planned using the DCM (Divergent Component of Motion), and the green line represents the position trajectory of the robot’s COM (center of mass). From the figures, it can be observed that the error between the planned trajectory and the actual trajectory of the robot is controlled within 2 cm, indicating the high precision of the proposed control model. Figure 14 and Figure 15 describe the variation of the center of mass velocity during the walking process in both the simulation and physical experiments. The change in center of mass velocity during the transition between the swing leg and the support leg is smooth, achieving the expected experimental results.

### 5.2. Forward Walking

**Figure 16:**
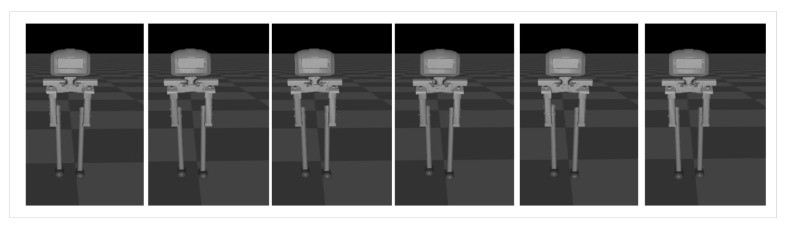
Screenshots of the L03 robot’s forward walking in the simulation experiment.

**Figure 17:**
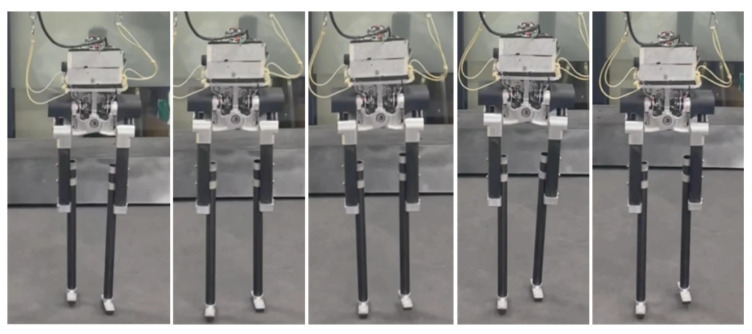
Screenshots of the actual L03 robot’s forward walking experiment.

Forward walking refers to the dynamic forward movement of a robot during locomotion. In this study, we applied a control algorithm for forward walking to the L03 robot and conducted an analysis of its stability during the walking process. To assess the stability of the robot in forward walking, both simulation and physical experiments were performed to evaluate its performance. Figure 16 and Figure 17 illustrate the forward walking process of the robot in the simulation and physical experiments, respectively. The experimental results demonstrate the robot’s ability to achieve stable forward walking.

**Figure 18:**
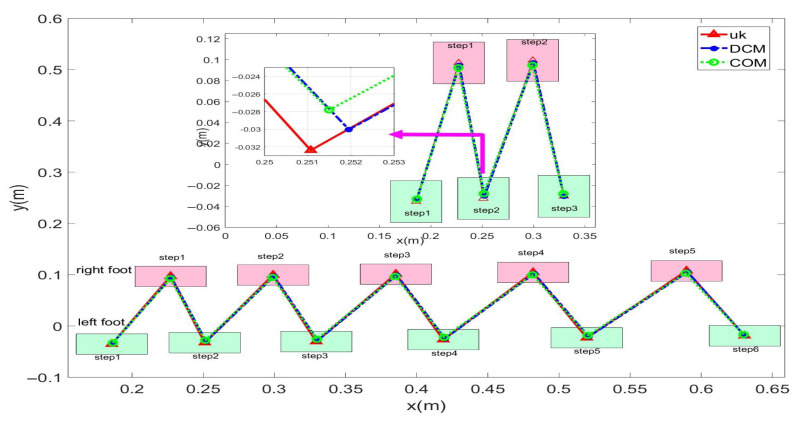
Simulation data for the L03 robot’s forward walking.

**Figure 19:**
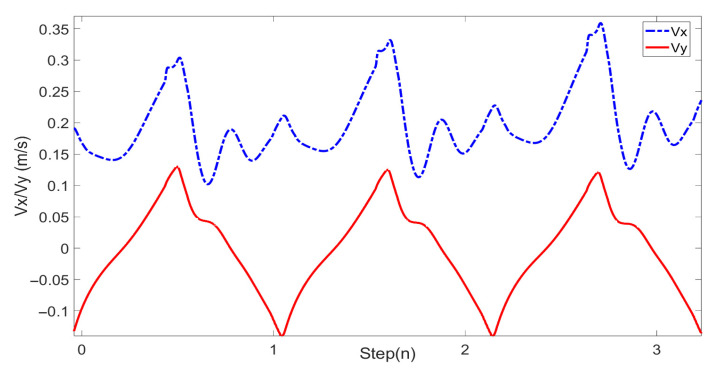
COM velocity profile of the L03 robot’s forward walking during the simulation.

**Figure 20:**
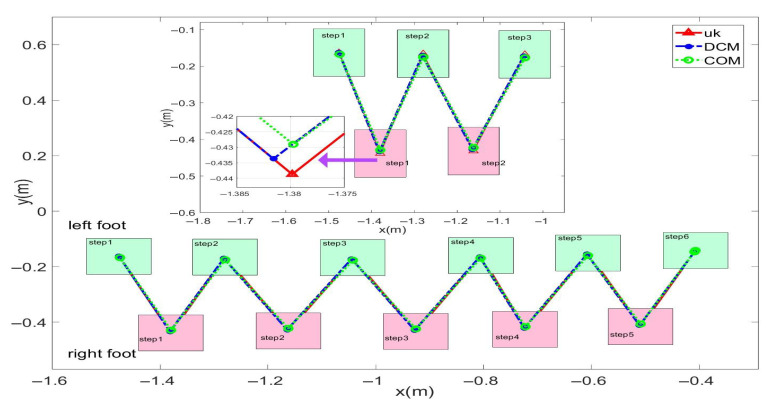
Experimental data for the L03 robot’s forward walking.

**Figure 21:**
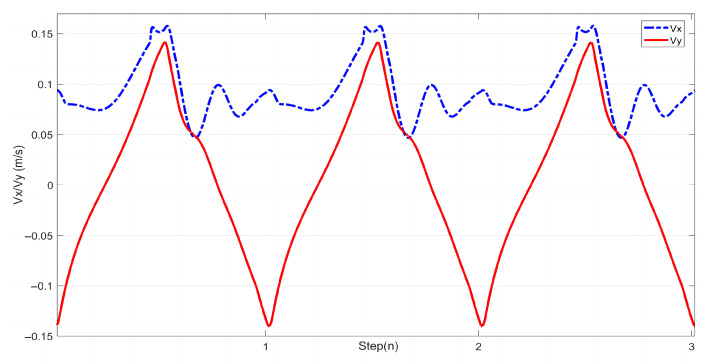
COM velocity profile of the L03 robot’s forward walking during the experiment.

In this study, forward walking experiments with the L03 robot were conducted in both the MuJoCo environment and real-world scenarios; the results of the simulation are shown in Figure 18 and Figure 19, while the results of the physical experiments are shown in Figure 20 and Figure 21. A speed of 0.3 m/s and a step length of 0.07 m were set in the MuJoCo simulation experiment, while in the real-world walking experiment a speed of 0.3 m/s and a step length of 0.2 m were used. All experiments were conducted on flat ground. Figure 16 and Figure 17 illustrate the walking gaits of the robot in the simulation and physical experiments, respectively. The graphs show the actual footstep position trajectory in red, the footstep position trajectory planned by DCM in blue, and the position trajectory of the robot’s COM in green. It can be observed from the figures that the error between the planned trajectory and the actual trajectory of the robot is controlled within 2 cm, indicating the high precision of the proposed control model and the robot’s good robustness. Figure 18 and Figure 20 describe the variation of the center of mass velocity during the walking process in the simulation and physical experiments, respectively. The change in the center of mass velocity during the transition between the swing leg and the support leg is smooth, achieving the expected experimental results.

### 5.3. Stability Analysis

In order to further test the stability of the L03 robot, a series of experiments were designed in this study, including a leg impact experiment, torso strike experiment, and foot slip experiment. Foam padding was added to the robot’s torso to protect it from damage, increasing its overall weight to 6.2 kg and height to 1.12 m. In the leg impact experiment, a fitness ball with a diameter of 65 cm and a weight of 885 g was used, resulting in a mass ratio of 14.2% compared to the robot. For the strike experiment, a lightweight volleyball with a diameter of 65 cm and a weight of 260 g was used, resulting in a mass ratio of 4.1% compared to the robot. In the foot slip experiment, carbon fiber tubes with a diameter of 10 mm were used as ground obstacles. For each experiment, qualitative analysis was conducted to study the robot’s response.

**Figure 22:**
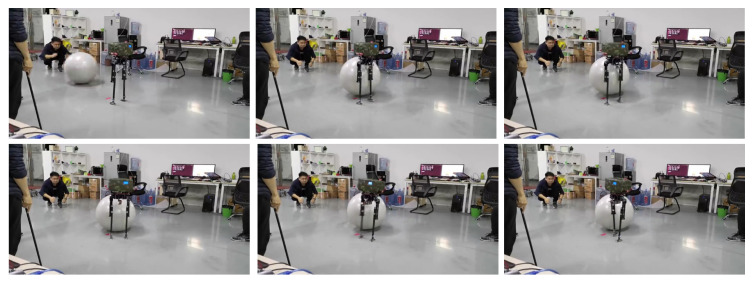
Video screenshots of the leg impact experiment.

As shown in Figure 22, the L03 robot exhibited the following behaviors in the leg impact experiment: during walking, the right swing leg was impacted before landing, resulting in the robot spinning; to maintain balance, the robot quickly lifted its left leg and took a large step forward, followed by the right leg taking a small step backward. By adjusting its foot placement, the robot successfully regained its balanced state of stationary stepping.

**Figure 23:**
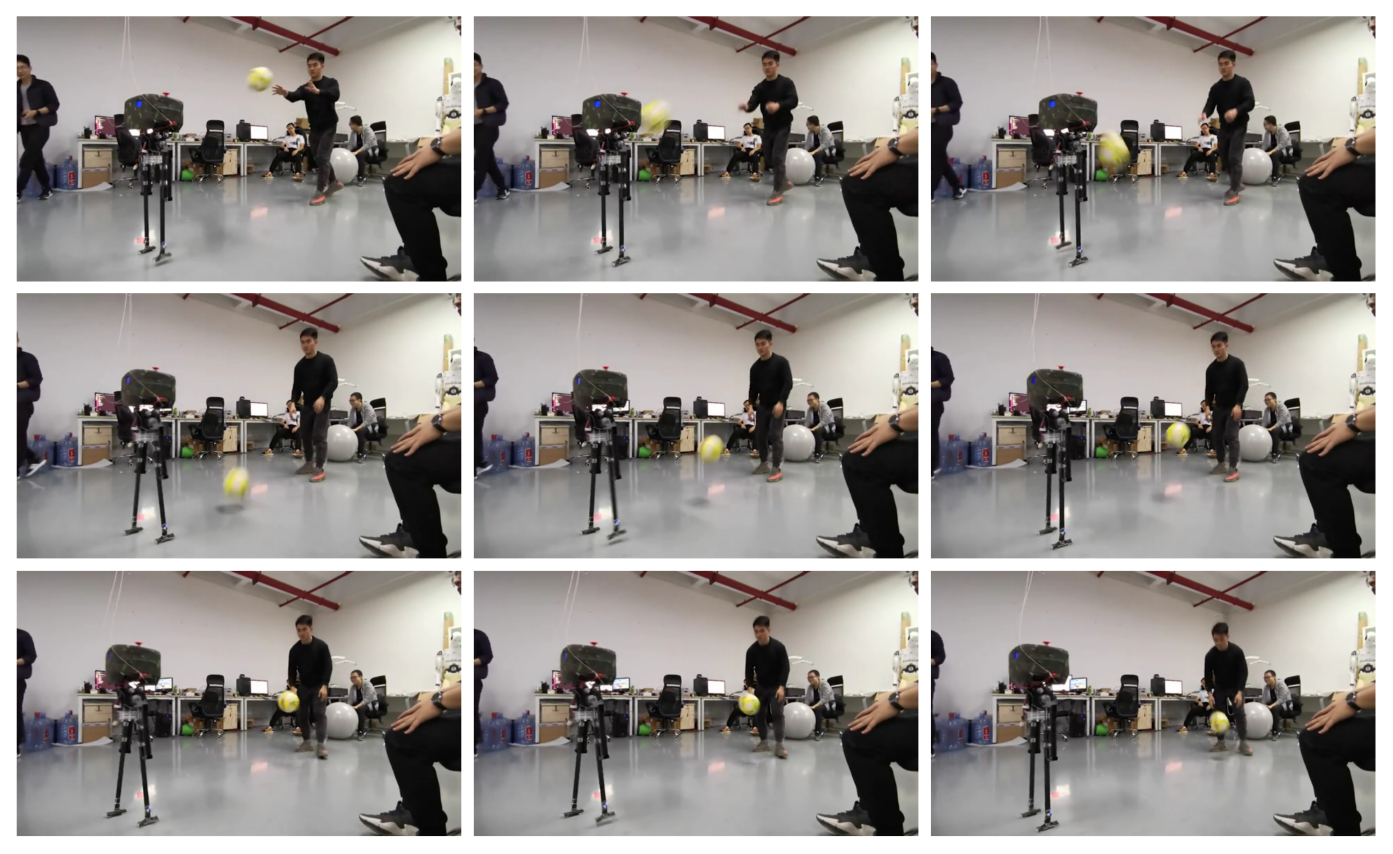
Video screenshots of the trunk impact experiment.

As shown in Figure 23, in the trunk impact experiment the L03 robot was struck by a volleyball and attempted to rebound while performing stationary stepping. The impact caused the robot to lean backward. To maintain stability, the robot’s left swing leg quickly took a large step backward, followed by the right swing leg taking a small step backward to maintain balance. Subsequently, the left swing leg continued to take a large step backward, while the right swing leg lightly touched the ground. The robot remained in this state until the impact force dissipated completely, returning to its original stable stepping condition.

**Figure 24:**
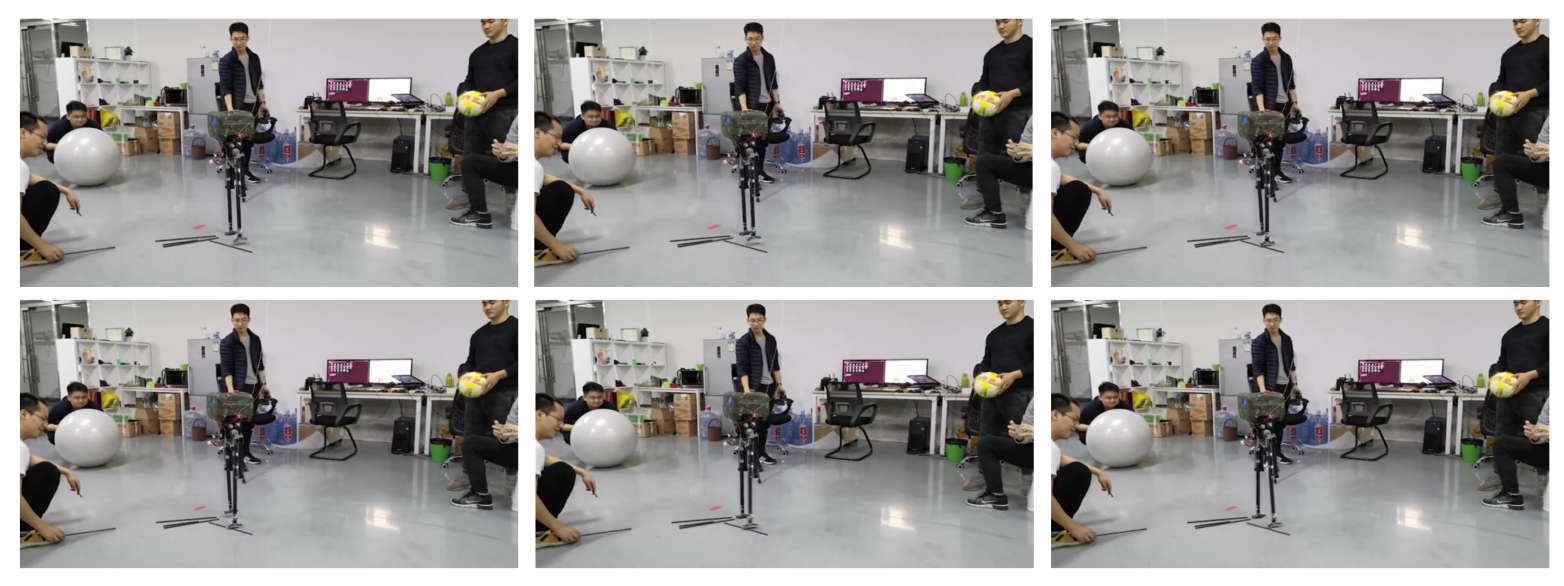
Video screenshots of the foot slippage experiment.

In the foot slip experiment, the robot stepped on a cylindrical tube that caused the robot’s feet to slip, disturbing its balance. As shown in Figure 24, during the stationary stepping of the robot, the right swing leg stepped on the small cylindrical tube, causing the robot’s foot placement to slide and resulting in a forward tilt. To restore balance, the left swing leg quickly swung forward and touched the ground, while the right swing leg took a small step forward, allowing the robot to regain its balanced state. Due to the minor nature of the disturbance caused by foot slipping, only two steps were required for the robot to successfully readjust.

## 6. Conclusions and Discussion

This research aimed to investigate the minimum number of actuators required to achieve full omnidirectional walking in a bipedal robot, drawing inspiration from the segmented design approach used in passive walking robots. By implementing a segmented design for the lateral hip joints, we were able to further reduced the number of actuators. To accomplish this, we introduce a bipedal robot named L03, equipped with only five actuators. Our approach is based on theoretical derivation of the kinematics and dynamics coupled with advanced control algorithms for DCM-based motion planning and trajectory tracking using a linear inverted pendulum model. Through simulations and experiments, we demonstrate that the bipedal L03 robot is capable of simple walking with a certain degree of disturbance rejection. This indicates that the segmented design approach can be applied to active walking robots, providing a foundation for future development of a bipedal robot with a segmented design for the anterior–posterior hip joints, enabling full omnidirectional walking with just four actuators. Overall, the distinctive structural design of the L03 bipedal robot significantly reduces the weight and leg rotational inertia, making it closely resemble the characteristics of the linear inverted pendulum model. This design enables the swift transplantation of theoretical algorithms for validation with the physical robot. Additionally, owing to its mere five actuators, the L03 robot’s simple structure and low cost make it an ideal platform for studying bipedal walking algorithms. This research holds crucial significance for the further advancement and practical application of algorithms in bipedal robots. With the growing integration of AI techniques in bipedal robot gait control, the low number of actuators in the L03 bipedal robot facilitates seamless integration with AI, further amplifying the exceptional hardware platform provided by such robots. In the future, our team will continue to explore algorithmic research on the L03 platform and investigate the application of artificial intelligence in the development of bipedal robot control algorithms.

## Figures and Tables

**Table 1:** Description of robot parameters.

Parameter	Quantitative Value	
length	/	0.284 m
width	/	0.16 m
height	normal	0.92 m
	highest	0.94 m
	lowest	0.84 m
weight	total	5.5 kg
	hip	4.41 kg
	leg	0.68 kg
lateral hip	range	−10° to 10°
	peak velocity	155°/s
	peak torque	195 Nm
pitch hip	range	−90° to 90°
	peak velocity	540°/s
	peak torque	56 Nm
slide leg	leg length range	580 mm to 680 mm
	peak velocity	1.5 m/s
	peak force	1120 N

The rated rotational speed of the Maxon DC motor RE35 is 4800 rpm, with a rated torque of 0.108 Nm and a peak torque of 1.12 Nm.

## Data Availability

Not applicable.
